# Human Colon Microbiota Transform Polycyclic Aromatic Hydrocarbons to Estrogenic Metabolites

**DOI:** 10.1289/ehp.7259

**Published:** 2004-09-22

**Authors:** Tom Van de Wiele, Lynn Vanhaecke, Charlotte Boeckaert, Kerry Peru, John Headley, Willy Verstraete, Steven Siciliano

**Affiliations:** ^1^Laboratory of Microbial Ecology and Technology (LabMET), Ghent University, Gent, Belgium; ^2^National Water Research Institute, Environment Canada, Saskatoon, Saskatchewan, Canada; ^3^Department of Soil Research, University of Saskatchewan, Saskatoon, Saskatchewan, Canada

**Keywords:** aryl hydrocarbon receptor, estrogen receptor, oral exposure, simulator of the human intestinal microbial ecosystem (SHIME)

## Abstract

Ingestion is an important exposure route for polycyclic aromatic hydrocarbons (PAHs) to enter the human body. Although the formation of hazardous PAH metabolites by human biotransformation enzymes is well documented, nothing is known about the PAH transformation potency of human intestinal microbiota. Using a gastrointestinal simulator, we show that human intestinal microbiota can also bioactivate PAHs, more in particular to estrogenic metabolites. PAH compounds are not estrogenic, and indeed, stomach and small intestine digestions of 62.5 nmol naphthalene, phenanthrene, pyrene, and benzo(*a*)pyrene showed no estrogenic effects in the human estrogen receptor bioassay. In contrast, colon digests of these PAH compounds displayed estrogenicity, equivalent to 0.31, 2.14, 2.70, and 1.48 nmol 17α-ethynylestradiol (EE2), respectively. Inactivating the colon microbiota eliminated these estrogenic effects. Liquid chromatography–mass spectrometry analysis confirmed the microbial PAH transformation by the detection of PAH metabolites 1-hydroxypyrene and 7-hydroxybenzo(*a*)pyrene in colon digests of pyrene and benzo(*a*)pyrene. Furthermore, we show that colon digests of a PAH-contaminated soil (simulated ingestion dose of 5 g/day) displayed estrogenic activity equivalent to 0.58 nmol EE2, whereas stomach or small intestine digests did not. Although the matrix in which PAHs are ingested may result in lower exposure concentrations in the gut, our results imply that the PAH bioactivation potency of colon microbiota is not eliminated by the presence of soil. Moreover, because PAH toxicity is also linked to estrogenicity of the compounds, the PAH bioactivation potency of colon microbiota suggests that current risk assessment may underestimate the risk from ingested PAHs.

Polycyclic aromatic hydrocarbons (PAHs) are high-priority environmental contaminants because of their toxic, carcinogenic, and putative estrogenic or antiestrogenic properties in the human body. Human exposure to high-molecular-weight PAHs mainly occurs through oral uptake of charcoal-broiled, grilled, and smoked meats (van Maanen et al. 1994) and through ingestion of soil or poorly cleaned vegetables, resulting in exposed doses about an order of magnitude higher than exposure by inhalation (Heisterkamp and van Veen 1997). The hazardous effects of ingested PAHs come from the PAH fraction released from the nutrients, soil, or associated organic matter in the intestinal lumen and that, upon intestinal absorption, reaches the intestine enterocytes and liver hepatocytes. In these cells, PAHs may act as ligands to the human aryl hydrocarbon (Ah) receptor, which plays a central role in the toxic response of specific aromatic hydrocarbons by the regulation of typical human biotransformation enzymes (reviewed by Hankinson 1995).

The risk from orally ingested PAHs is currently thought to be reduced when co-ingested soil or fibers decrease the intestinal PAH absorption and hence bioavailability (De Kok and van Maanen 2000). Most ingested PAHs pass harmlessly through the gastrointestinal (GI) tract without being transformed by human enzymes to hazardous metabolites. However, this assumes that no microbial bio-transformation of PAHs occurs. The human GI tract harbors an incredibly diverse microbial community, which typically performs fermentative processes but which is also capable of transforming xenobiotic compounds (Aura et al. 2002; Ilett et al. 1990; Macdonald et al. 1983). Hence, if microbial PAH bio-transformation in the human colon is possible, the susceptibility of the colon epithelium to bioactive PAH metabolites may increase the health risks that are associated with non-absorbed PAHs that reach the colon. To date, no information is available on the PAH bio-activation potency from human colon microbiota. To evaluate this, in this study we investigated the estrogenicity of PAHs because several PAH metabolites structurally resemble steroidal hormones that bind the human estrogen receptor (ER) (Ariese et al. 2001), which could thus lead to estrogenic or anti-estrogenic activity *in vivo*.

We opted for an *in vitro* approach to specifically look for microbial biotransformations and thus avoid possible interference from colon epithelium enzymes that would be present in an *in vivo* approach. Pure PAH compounds and a PAH-contaminated urban soil were incubated in the stomach, small intestine, and colon suspensions from a simulator of the human GI tract. Given the aromaticity of PAHs, we used a modified Ah receptor yeast assay (Miller 1997) to investigate whether the PAHs in the different digests could activate the human Ah receptor and subsequently induce signal transduction. We also investigated the estrogenicity of the PAH incubated digests by monitoring activation of the human ER in a modified ER yeast assay (Routledge and Sumpter 1996). In addition, we applied a newly optimized liquid chromatography–mass spectrometry (LC-MS) protocol to detect whether PAH metabolites were formed during incubation.

## Materials and Methods

### Chemicals.

PAH parent compounds naphthalene, phenanthrene, pyrene, and benzo(*a*)pyrene (reagent grade) were obtained from Sigma-Aldrich (Bornem, Belgium). To avoid solubility problems in the incubation tests, PAHs were first dissolved in ethanol before digestion. All stock solutions were prepared in amber glass bottles and stored in the dark at 4°C. Hydroxy-PAH metabolites 1-OH naphthalene, 9-OH phenanthrene, 1-OH pyrene, and 7-OH benzo(*a*)pyrene were reagent grade and also obtained from Sigma-Aldrich.

### Incubations.

**Figure 1:**
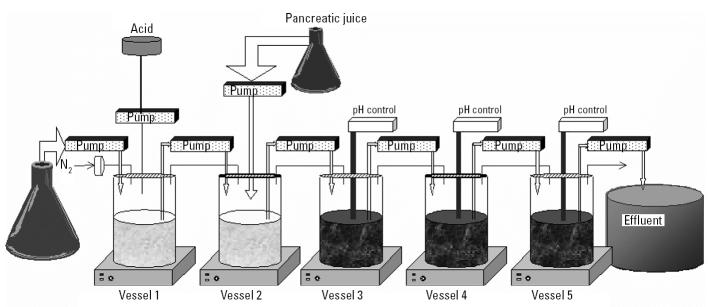
Schematic representation of SHIME. Vessels 1–5 simulate conditions from the stomach, small intestine, colon ascendens, colon transversum, and colon descendens, respectively.

PAHs and soils were incubated in batches by sampling GI suspensions from a simulator of the human intestinal microbial ecosystem (SHIME). This dynamic model of the GI tract consists of five compartments representing the stomach, small intestine, and colon ascendens, transversum, and descendens (Figure 1). The colon suspension contains *in vitro* cultured microbiota that were isolated from human feces and are representative of the *in vivo* colon microbial ecology after a growth stabilization period in the different colon compartments (Molly et al. 1993). A typical stomach digestion consists of an incubation of PAHs or PAH-contaminated soil samples for 3 hr at pH 1.5 at 37°C. A small intestine digestion consists of an incubation for 5 hr at pH 7 at 37°C in the presence of bile salts (0.2 mmol/L) and pancreatic enzymes supplemented as pancreatic powder of porcine origin (0.4 g/L). A colon digestion consists of an incubation with colon microbiota for 48 hr at 37°C, withdrawn from the colon vessels of the SHIME reactor. Some samples were incubated with inactive colon microbiota. For this, colon microbiota were autoclaved for 30 min (121°C, 1 bar overpressure). Incubation of PAH standard compounds in stomach, small intestine, and colon digests occurred at a concentration of 20 μmol/L. This concentration is normally not encountered in the GI tract but gave us more possibilities to study microbial PAH metabolism in depth. GI digestion experiments on soil samples were performed as previously described (Van de Wiele et al. 2004b) to simulate a hypothetical soil ingestion of 5 g/day by pica-afflicted children (stomach, 40 mL; small intestine, 60 mL; colon, 100 mL). To avoid photocatalytic effects, all digestions were performed in amber flasks. After the respective incubations, samples were centrifuged at 3,000*g* for 10 min to remove most of the particulates and biomass. The supernatants were then stored at −20°C before analysis.

### Sample treatment.

PAH parent components and PAH metabolites were extracted from the digests by performing a liquid/liquid extraction in which the digest and ethyl acetate were mixed in a 1:1 ratio. The ethyl acetate fraction was then put in a rotary evaporator to remove most of the solvent. The remainder of the solvent was removed under a gentle stream of nitrogen gas and finally replaced by dimethyl sulfoxide, which is suitable for use in bioassay tests. For chemical analysis of the samples using LC-MS, sample aliquots were subjected to solid-phase extraction using PrepSep C_18_ (250 mg; Fisher Scientific, Edmonton, Alberta, Canada). Hydroxy-PAHs were eluted with methanol.

### PAH conjugate analysis.

To check whether conjugated PAH metabolites were formed in the different digests, samples were also incubated in the presence of β-glucuronidase and aryl sulfatase, both obtained from Sigma-Aldrich. After the PAH parent compounds had been incubated in SHIME suspension, a 1 mL aliquot of these samples was diluted in 1 mL 0.1 M acetate buffer, and the pH was adjusted to 5 with sodium hydroxide. A volume of 400 μL β-glucuronidase (100 U/mL) and 250 μL aryl sulfatase (60 U/mL) was added, and the mixture was incubated for 6 hr at 37°C to hydrolyze the PAH conjugates.

### Bioassays.

For the bioassays, we used a modified protocol from De Boever et al. (2001) that was based on the protocol developed by Routledge and Sumpter (1996) for the yeast estrogen bioassay and Miller (1997) for the yeast Ah bioassay. Briefly, these researchers transformed *Saccharomyces cerevisiae* with the human Ah receptor gene and the human *ER-*α gene, together with expression plasmids containing responsive elements and the *lacZ* reporter gene (encoding the enzyme β-galactosidase). The expression of β-galactosidase is triggered by test chemicals, which upon binding to the respective receptors induce the conformational change necessary for binding of the receptor/ligand dimer to the responsive elements. This β-galactosidase activity is quantified at 540 nm by the conversion of the chromogenic substance chlorophenol red–β-d-galactopyranoside into chlorophenol red. The bioassay response is expressed as the absorbance at 540 nm divided by the optical density at 630 nm [(A540/A630)_net_]. Positive signals in the Ah receptor assay were typically expressed as percentage equivalence to 200 nM benzo(*a*)pyrene, which arbitrarily corresponded to a bioassay response of 100%. Similarly, estrogenic activity of the samples was expressed as percentage equivalence to 6.96 nM 17α-ethynyl estradiol (EE2), which elicited a 100% response in the ER bioassay (De Boever et al. 2001). To make sure that background signals from GI suspensions of soil or food matrices did not interfere with the detection of estrogenic signals in the bioassays, corrections were made in a set of negative control experiments by subtracting the response of a PAH-containing digest from that from a blank digest without PAHs (see Supplemental Material available online at http://ehp.niehs.nih.gov/docs/2004/7259/supplemental.pdf). The bio-assays were performed in 96-well plates in which 10 μL of the test compounds was tested and incubated with 240 μL of the genetically modified yeast (optical density, 0.25 at 610 nm). Serial dilutions of the test compounds were made in dimethyl sulfoxide, which allowed generation of dose–response curves for doses (ordinate) versus activity (abscissa). The data were fitted by a four-parametric logistic model using the Marquardt-Levenberg algorithm (Sigmaplot 4.0; SPSS Inc., Chicago, IL, USA) (De Boever et al. 2001).

### PAH analysis.

Sample treatment for and determination of PAHs were performed by the Environmental Research Centre (Erembodegem, Belgium). Briefly, PAHs from pellets were extracted by a 1:1 acetone:hexane mixture using an ASE 200 accelerated solvent extractor (Dionex, Sunnyvale, CA, USA). PAHs from supernatants were extracted with dichloromethane. Analysis of the PAH content in the extracts was performed according to a standardized method [U.S. Environmental Protection Agency (EPA) method 8270 (U.S. EPA 1996)] by gas chromatography coupled with mass spectrometry (GC-MS). We used a quadrupole mass spectrometer (Trace-MS; Fisons/Thermoquest, Antwerp, Belgium). The detection limit for the different PAH components was 0.2 μg/L, and the quantification limit was 0.4 μg/L. The extraction efficiency of the sample preparation step before PAH analysis was between 80 and 110%, as determined with reference soil CRM535 (Environmental Research Center, Erembodegem, Belgium).

### LC-MS analysis.

We performed LC-MS analysis of the samples for hydroxy-PAHs as previously described (Van de Wiele et al. (2004a). The identity of hydroxy-PAH metabolites in the samples was confirmed by using synthetic standards of these metabolites and comparing the HPLC profiles from the colon digests with those from the standards. Briefly, all samples for LC-MS analysis were subjected to solid-phase extraction using PrepSep C_18_ columns (250 mg). Sample volumes of 5 mL were loaded on the columns and washed with 10 mL Milli-Q water; the target analytes were then eluted with 10 mL methanol. Aliquots (1 mL) were subsampled and stored in amber vials at 4°C before LC-MS analysis. HPLC analysis was performed using a Waters 2695 separation module (Waters, Milford, MA, USA). The selected column was a 2.1 mm × 100 mm, 3.5 μm particle size, Waters XTerra MS C_18_ column, which was kept at a constant temperature of 26°C. The binary eluent system consisted of methanol:water 90:10 (vol/vol; eluent A) and methanol:water 10:90 (vol/vol; eluent B). MS analysis was performed with a Quattro Ultima Mass spectrometer (Micromass Technologies, Manchester, UK) that was equipped with an electrospray interface operating in the negative ion mode. Instrumental control and data acquisition were performed with MassLynx software version 3.5 (MicroMass Ltd., Manchester, UK). The electrospray ionization source was operated at 90°C, a desolvation temperature of 200°C, a cone voltage of 61 V, and a capillary voltage of 2.74 kV. Nitrogen gas served as the cone gas (flow rate of 159 L/hr), desolvation gas (490 L/hr), and nebulizer gas (set to maximum). The detector multiplier voltage was set to 650 V. We used selected ion monitoring for quantitative analysis monitoring the (M – H)^−^ of *m*/*z* for the hydroxy-PAHs.

## Results and Discussion

**Figure 2:**
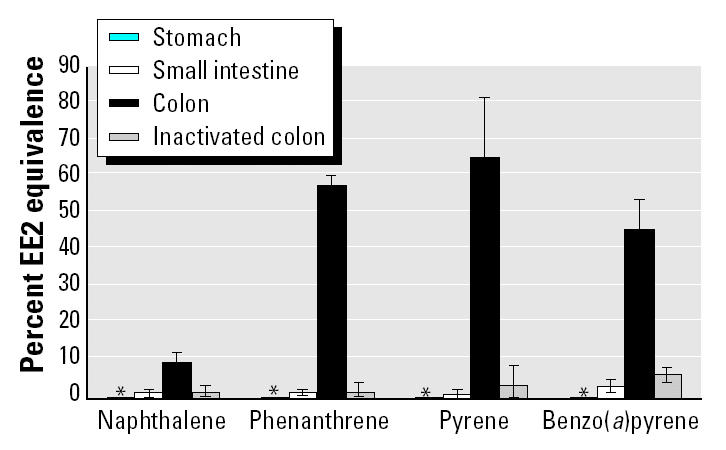
Estrogen response (mean ± SD) of naphthalene, phenanthrene, pyrene, and benzo(*a*)pyrene (62.5 nmol/L) incubated in stomach, small intestine, and colon digests and in digests with inactivated colon microbiota. Values are means of four replicates.
* None of the stomach digestions gave a significant response in the estrogen bioassay.

Because of their moderate-to-high degree of aromaticity, we expected pure solutions of naphthalene, phenanthrene, pyrene, and benzo(*a*)pyrene to test positive in the Ah bioassay. Naphthalene (200 nM) displayed 0.4% benzo(*a*)pyrene equivalence, whereas 200 nM phenanthrene and 200 nM pyrene displayed 15.1 and 48.2% benzo(*a*)pyrene equivalence, respectively. PAH compounds are not estrogenic, and indeed, up to 16 μM of the four pure PAHs did not induce an estrogenic response in the estrogen bioassay. Similarly, separate stomach and small intestine digests of the four PAHs did not show a significant estrogen response (Figure 2). In contrast, PAHs from colon digests became estrogenic. Conversion of the percent EE2 equivalence values, shown in Figure 2, to equivalent EE2 concentrations is as follows: for colon digests of 62.5 nM pyrene, 2.70 nM EE2 equivalence; for phenanthrene, 2.14 nM EE2 equivalence; for benzo(*a*)pyrene, 1.48 nM EE2 equivalence; and for naphthalene, 0.31 nM EE2 equivalence. This PAH bio-activation was only evident in colon digestion. This shows the selectivity of colon digestion toward an increase in estrogenicity, whereas no increased Ah response was detected, compared with stomach or small intestine digests. To make sure that the observed effects were not coming from the matrix background of the colon interacting with PAHs, we incubated PAHs in a heat-inactivated colon suspension. The removal of microbial activity markedly reduced the increase in estrogenic activity (Figure 2). This finding indicates that the risk for PAH bioactivation along the GI tract is not exclusively associated with human bio-transformation enzymes from the enterocytes in the small intestine epithelium and colonocytes in the large intestine epithelium (Autrup et al. 1978; De Kok and van Maanen 2000; Doherty and Charman 2002), but that colon microbiota can also bioactivate PAHs.

**Figure 3:**
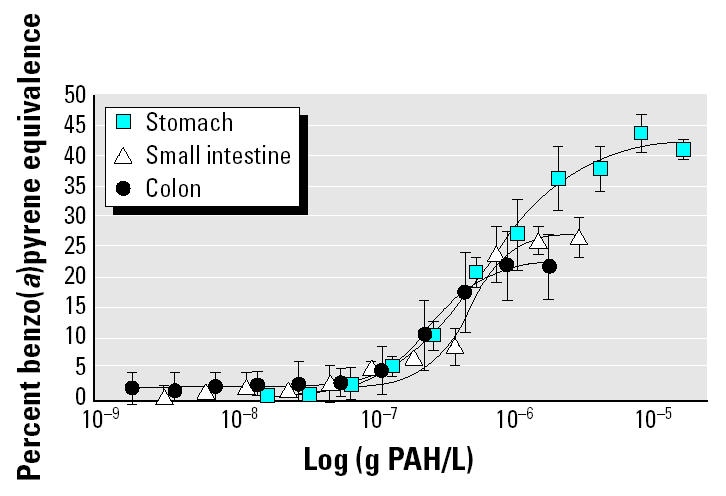
Dose–response curve of stomach, small intestine, and colon digests of PAH-contaminated playground soil in the Ah receptor yeast bioassay, expressed as percent benzo(*a*)pyrene equivalence in function of released PAH concentrations in the respective digests. Values are means of four replicates; error bars represent SD.

**Figure 4:**
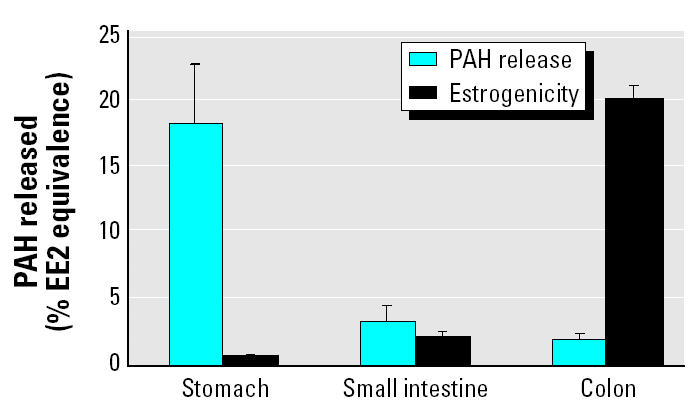
Released concentrations of PAHs and estrogen response in stomach, small intestine, and large intestine digests incubated with PAH-contaminated soil samples. Values are means of four replicates; error bars represent SD.

We then evaluated the significance of this process using lower, more realistic concentrations obtained from soil from a former urban playground contaminated with 49 ± 1.5 mg PAHs per kilogram soil (dry weight) by years of atmospheric deposition. Pica-afflicted children form the largest risk group for soil ingestion because of their unusual hand–mouth behavior and low body weight. Hence, we simulated the GI tract of a pica child, hypothetically ingesting 5 g soil/day. GC-MS analysis previously showed that the released PAH fraction from the soil matrix was highest in the stomach digest (18 ± 5.3 μg/L), followed by the small intestine digest (3 ± 1.1 μg/L) and the colon digest (2 ± 0.3 μg/L) (Van de Wiele et al. 2004b). This corresponded to a maximal Ah bioassay response for the stomach digest of 41 ± 2.9% benzo(*a*)pyrene equivalence; the small intestine, 27 ± 1.4%; and the colon, 22 ± 2.6% (Figure 3). Based on the role of the human Ah receptor in the toxicity of specific aromatic hydrocarbons, these findings would normally indicate that the colon digest represents the lowest risk for PAH bioactivation. Surprisingly, the trend in estrogenic activity was the inverse of observed PAH release or Ah bioassay response. Similar to the estrogen bioassay results on pure PAHs, there was negligible induction of estrogenic activity in the stomach (0.6%) and small intestine (2.0%) digestion (Figure 4). However, an average value of 20.1 ± 0.84% EE2 equivalence was observed in a colon digestion of the contaminated soil (Figure 4). We infer that the PAH bioactivation potency from colon microbiota also occurs at lower and relevant concentrations for human exposure and that the presence of soil does not eliminate this potency.

Soil organic matter and nutritional fibers are known to lower the fraction of a contaminant that can be absorbed by the intestine (O’Neill et al. 1991; Oomen et al. 2000; van Schooten et al. 1990). This would theoretically lower the risk from ingested contaminants because bioactivation by human biotransformation enzymes will be reduced because of a lower bioavailability. To test this hypothesis, we compared the estrogenicity from colon-incubated PAHs in the presence and absence of soil by calculating the bioactivation potency of the digests as estrogenicity/aromaticity. We divided—at equimolar concentrations—the percent EE2 equivalence of the different digests by their respective percent benzo(*a*)pyrene equivalence. At equimolar concentrations of 8.03 nmol PAH/L, this ratio was 0.93 for naphthalene, 2.16 for phenanthrene, 0.98 for pyrene, and 0.12 for benzo(*a*)pyrene, whereas the colon digest of the PAH-contaminated soil gave a ratio of 0.88, the same order of magnitude as the ratios for pure PAH compounds and one order of magnitude higher than the ratios for the stomach soil digest (0.016) or small intestine soil digest (0.077). These findings provide further evidence that the presence of a soil matrix does not eliminate the PAH bioactivation by colon micro-biota and that the estrogenic potency of soil-derived PAHs does not significantly decrease if compared with pure PAHs.

PAH metabolites that typically have estrogenic properties are hydroxylated derivatives because of their structural similarity to natural estrogens (Fertuck et al. 2001; Hirose et al. 2001). Hence, in the next step of the research, we screened with LC-MS for the presence of hydroxy-PAHs by analyzing the respective colon digests of 20 μmol/L pure PAH compounds. The identity of PAH metabolites was confirmed by comparing the HPLC profiles and MS spectra of the colon digests with those from chromatographic synthetic standards of several hydroxy-PAHs. The developed protocol had reasonably low detection limits for 1-OH naphthalene, 9-OH phenanthrene, 1-OH pyrene, and 7-OH benzo(*a*)pyrene (Table 1) (Van de Wiele et al. 2004a). No hydroxy-PAHs were detected upon stomach or small intestine incubations. From all colon digests, only the pyrene digest tested positive for a hydroxy-PAH metabolite, with 1-OH pyrene at a concentration of 2.5 μg/L (Table 1). Glucuronidated or sulfated PAH conjugates are also typical bio-transformation products from eukaryotic organisms (Cajthaml et al. 2002). Because the concentration of fungi and yeasts in the colon suspension amounted to 4.3 ± 0.6 log colony forming units (CFU)/mL, we tested whether PAH conjugates were present in colon digests of pure PAH compounds. Glucuronidase and arylsulfatase typically cleave off glucuronic acid or sulfate groups from conjugated PAHs, regenerating the hydroxy-PAH metabolites (Cajthaml et al. 2002). After incubating the extracts of the colon digests in the presence of glucuronidase (100 U/mL) and arylsulfatase (60 U/mL) for 6 hr at 37°C, we found higher concentrations of 1-OH pyrene (4.4 μg/L) and a new metabolite, 7-OH benzo(*a*)pyrene (1.9 μg/L). No hydroxy-PAHs were retrieved from inactivated colon samples. Although other hydroxy-PAHs may have formed than those tested during LC-MS analysis, these analytical data show that PAH bioactivation by colon microbiota may result from hydroxy-PAH metabolites.

The formation of hydroxy-PAH metabolites and especially the increased estrogenicity by human colon microbiota bring up two questions: are the observed transformations plausible for the *in vivo* human GI tract, and to what extent can bioactive PAH metabolites contribute to the total risk from oral PAH exposure? To answer the first question, literature shows that resident gut microbiota may influence xenobiotic metabolism from the intestinal epithelium (Hooper et al. 2001). Additionally, microbial glucuronidase activity in the intestine sometimes cleaves off glucuronic acid groups from excreted human conjugated metabolites, thus regenerating the more bioactive hydroxylated intermediates (Aura et al. 2002). These reports describe indirect effects of intestinal microbiota toward xenobiotic metabolism. However, our findings indicate a direct metabolism effect of human colon microbiota toward PAH parent compounds, because the *in vitro* approach used in the present study eliminated possible interferences by intestinal epithelium enzymes. The observed biotransformation and bioactivation reactions originate from a microbial community that resembles that of the *in vivo* intestinal lumen both in composition and in metabolic activity. Rather than containing the less active microbiota from fecal matter, the microbial community from the used *in vitro* method is more representative of the different parts of the human colon (Molly et al. 1993).

As suggested by the LC-MS results, the colon microbiota formed hydroxy-PAH metabolites, which may seem unlikely because this oxidative step would occur in an anaerobic environment, as shown by redox potential values from the colon suspension, which varied between −180 mV and −230 mV. These values are well within the range of −145 mV to −250 mV reported for the colon *in vivo* (Bowler et al. 2001; Chourasia and Jain 2003). Yet, oxidative reactions by intestinal bacteria from humans, mice, and rats have been described for the conversion of 2-amino-3-methylimidazo[4,5-*f* ]quinoline to its reportedly mutagenic 7-keto derivative (Rumney et al. 1993). Additionally, *Enterococcus faecalis* even performs aromatic hydroxylation reactions in the intestine *in vivo* (Huycke and Moore 2002). It is therefore not unlikely that intestinal microbiota may hydroxylate PAHs, also given the fact that anaerobic PAH hydroxylation has been reported by microorganisms, albeit in sediments (Karthikeyan and Bhandari 2001). These studies on oxidative reactions by intestinal microbiota and anaerobic PAH biotransformations may thus support our findings, which need further study to identify which microorganisms bioactivate PAHs.

To answer the second question concerning the contribution of the observed effects to the total risk from PAH ingestion, further research is warranted. Yet, microbial PAH bioactivation to estrogenic metabolites may constitute an increased health risk when the human body is orally exposed to contaminated soils. Human colon epithelium is 20% more permeable to 17β-estradiol than is the human small intestine epithelium (van der Bijl and van Eyk 2003) and also has a higher permeability to hydrophobic compounds in general (Ungell et al. 1998). PAH metabolites with structures resembling steroidal hormones may thus exhibit weak estrogenic or antiestrogenic activity *in vivo* (Ariese et al. 2001). Because PAHs that reach the colon will be biotransformed by colon microbiota, we conclude that, in the *in vivo* situation, the colonic epithelium—which has ERs—may be subjected to hazardous effects from microbial PAH metabolites. The equivalent EE2 response of 20% for the colon-incubated environmental sample (Figure 4) indicates that the observed activation of the human ER is significant. Still, it must be kept in mind that a positive response in ER-reporter gene assays such as that from the present study does not necessarily predict endogenous transcription (Gozgit et al. 2004). Gozgit et al. (2004) noted that several PAHs induced activity in ER-reporter gene assays but that these PAHs did not up-regulate estrogen-responsive genes. The authors concluded that ER-reporter gene assays may detect concentrations of toxicants that are not physiologically active. In light of these recent findings, the estrogenic response from microbial PAH bioactivation in this study needs careful interpretation. However, the finding of 1-OH pyrene and 7-OH benzo(*a*)pyrene as metabolites from human colon microbiota is something that is not anticipated from current scientific knowledge or risk assessment studies. Comparison of our findings from active to those from inactivated colon microbiota shows that the microbial bioactivation potency is a factor of 12 higher than would be currently expected in risk calculations. Moreover, the time during which bioactive hydroxy-PAHs could react with colonocytes is also considerably longer (up to 72 hr) than the residence time in human enterocytes or hepatocytes (6 hr maximum). Additionally, if taken up by colonocytes, the hydroxy-PAHs are typical metabolites that are more easily metabolized by human biotransformation enzymes to, for example, potent carcinogens such as benzo(*a*)pyrene-r7,t8-dihydrodiol-t9,10-epoxide (Kim et al. 1998). Clearly, these literature reports on human PAH metabolism and our findings of PAH bioactivation by colon microbiota indicate the importance of conducting future work in which the relative importance of the human bioactivation processes versus the microbial bioactivation processes should be compared.

## Conclusion

Our results reveal that human colon microbiota can directly bioactivate PAHs, a potency that has not been reported before. As indicated by the analysis of a PAH-contaminated environmental sample, we also show that the presence of soil does not eliminate this microbial bioactivation potency. We therefore conclude that risk calculations that are based solely on human biotransformation enzymes may underestimate the risk from ingested aromatic contaminants because it does not consider the bioactivation processes described here.

## Figures and Tables

**Table 1:** Limits of detection (LODs) of hydroxy-PAHs obtained with LC-MS analysis, concentrations of the hydroxy-PAHs in the colon digest, and concentrations of the hydroxy-PAHs in the deconjugated colon digest.

Hydroxy-PAH	LOD (μg/L)	Colon digest (μg/L)	Colon digest deconjugated (μg/L)
1-OH naphthalene	7.33	ND	ND
9-OH phenanthrene	0.47	ND	ND
1-OH pyrene	0.24	2.5	4.4
7-OH benzo(*a*)pyrene	0.95	ND	1.9

ND, not detected.

## References

[b1-ehp0113-000006] Ariese F, Ernst WHO, Sijm DTHM (2001). Natural and synthetic organic compounds in the environment—a symposium report. Environ Toxicol Pharmacol.

[b2-ehp0113-000006] Aura AM, O’Leary KA, Williamson G, Ojala M, Bailey M, Puupponen-Pimia R (2002). Quercetin derivatives are deconjugated and converted to hydroxyphenylacetic acids but not methylated by human fecal flora in vitro. J Agr Food Chem.

[b3-ehp0113-000006] Autrup H, Harris CC, Trump BF, Jeffrey AM (1978). Metabolism of benzo(*a*)pyrene and identification of the major benzo(*a*)pyrene–DNA adducts in cultured human colon. Cancer Res.

[b4-ehp0113-000006] Bowler DG, Duerden BI, Armstrong DG (2001). Wound microbiology and associated approaches to wound management. Clin Microbiol Rev.

[b5-ehp0113-000006] Cajthaml T, Moder M, Kacer P, Sasek V, Popp P (2002). Study of fungal degradation products of polycyclic aromatic hydrocarbons using gas chomatography with ion trap mass spectrometry detection. J Chromatogr A.

[b6-ehp0113-000006] Chourasia MK, Jain SK (2003). Pharmaceutical approaches to colon targeted drug delivery systems. J Pharm Pharm Sci.

[b7-ehp0113-000006] De Boever P, Demare W, Vanderperren E, Cooreman K, Bossier P, Verstraete W (2001). Optimization of a yeast estrogen screen and its applicability to study the release of estrogenic isoflavones from a soygerm powder. Environ Health Perspect.

[b8-ehp0113-000006] De Kok TM, van Maanen JM (2000). Evaluation of fecal mutagenicity and colorectal cancer risk. Mutat Res.

[b9-ehp0113-000006] Doherty MM, Charman WN (2002). The mucosa of the small intestine. How clinically relevant as an organ of drug metabolism. Clin Pharmacokinet.

[b10-ehp0113-000006] Fertuck KC, Kumar S, Sikka HC, Matthews JB, Zacharewski TR (2001). Interaction of PAH-related compounds with the α and βisoforms of the estrogen receptor. Toxicol Lett.

[b11-ehp0113-000006] Gozgit JM, Nestor KM, Fasco MJ, Pentecost BT, Arcaro KF (2004). Differential action of polycyclic aromatic hydrocarbons on endogenous estrogen-responsive genes and on a transfected estrogen-responsive reporter in MCF-7 cells. Toxicol Appl Pharmacol.

[b12-ehp0113-000006] Hankinson O (1995). The aryl hydrocarbon receptor complex. Annu Rev Pharmacol Toxicol.

[b13-ehp0113-000006] Heisterkamp SH van Veen MP 1997. Exposure to Xenobiotics in Nutrition. Model Compounds: Butyl Benzyl Phthalate (BBP), Benzo[*a*]pyrene and Fluoranthene. Technical Report No. 604502 002. Bilthoven, Netherlands:RIVM.

[b14-ehp0113-000006] Hirose T, Morito K, Kizu R, Toriba A, Hayakawa K, Ogawa S (2001). Estrogenic/antiestrogenic activities of benzo(*a*)pyrene monohydroxy derivatives. J Health Sci.

[b15-ehp0113-000006] Hooper LV, Wong MH, Thelin A, Hansson L, Falk PC, Gordon JI (2001). Molecular analysis of commensal host-microbial relations hips in the intestine. Science.

[b16-ehp0113-000006] Huycke MM, Moore DR (2002). *In vivo* production of hydroxyl radical by *Enterococcus faecalis* colonizing the intestinal tract using aromatic hydroxylation. Free Radical Biol Med.

[b17-ehp0113-000006] Ilett KF, Tee LB, Reeves PT, Minchin RF (1990). Metabolism of drugs and other xenobiotics in the gut lumen and wall. Pharmacol Therapeut.

[b18-ehp0113-000006] Karthikeyan R, Bhandari A (2001). Anaerobic biotransformation of aromatic and polycyclic aromatic hydrocarbons in soil microcosms: a review. J Hazard Subst Res.

[b19-ehp0113-000006] Kim JH, Stansbury KH, Walker NJ, Trush MA, Strickland PT, Sutter TR (1998). Metabolism of benzo(*a*)pyrene and benzo(*a*)pyrene-7,8-diol by human cytochrome P450 1B1. Carcinogenesis.

[b20-ehp0113-000006] Macdonald IA, Mader JA, Bussard RG (1983). The role of rutin and quercitin in stimulating flavonol glycosidase activity by cultured cell-free microbial preparations of human feces and saliva. Mutat Res.

[b21-ehp0113-000006] Miller CA (1997). Expression of the human aryl hydrocarbon receptor in yeast. J Biol Chem.

[b22-ehp0113-000006] Molly K, Vandewoestijne M, Verstraete W (1993). Development of a 5-step multichamber reactor as a simulation of the human intestinal microbial ecosystem. Appl Microbiol Biot.

[b23-ehp0113-000006] O’Neill IK, Goldber MT, Ghissassi FE, Rojas-Moreno M (1991). Dietary fiber, fat and beef modulation of colonic nuclear aberrations and microcapsule-trapped gastrointestinal metabolites of benzo(*a*)pyrene-treated C57rB6 mice consuming human diets. Carcinogenesis.

[b24-ehp0113-000006] Oomen AG, Sips AJAM, Groten JP, Sijm DTHM, Tolls J (2000). Mobilization of PCBs and lindane from soil during *in vitro* digestion and their distribution among bile salt micelles and proteins of human digestive fluid and the soil. Environ Sci Technol.

[b25-ehp0113-000006] Routledge EJ, Sumpter JP (1996). Estrogenic activity of surfactants and some of their degradation products assessed using a recombinant yeast screen. Environ Toxicol Chem.

[b26-ehp0113-000006] Rumney CJ, Rowland IR, O’Neill IK (1993). Conversion of IQ to 7-OHIQ by gut microflora. Nutr Cancer.

[b27-ehp0113-000006] Ungell AL, Nylander S, Bergstrand S, Sjoberg A, Lennernas H (1998). Membrane transport of drugs in different regions of the intestinal tract of the rat. J Pharm Sci.

[b28-ehp0113-000006] U.S. EPA 1996. Method 8270C: Semivolatile Organic Compounds by Gas Chromatography/Mass Spectroscopy. In: Test Methods for Evaluating Solid Waste: Physical/Chemical Methods, Vol 1B. 3rd ed, Revision 3. Washington, DC:U.S. Environmental Protection Agency, Office of Solid Wastes.

[b29-ehp0113-000006] van der Bijl P, van Eyk AD (2003). Comparative in vitro permeability of human vaginal, small intestinal and colonic mucosa. Int J Pharm.

[b30-ehp0113-000006] Van de Wiele TR, Peru K, Verstraete W, Siciliano SD, Headley JV (2004a). Liquid chromatography mass spectrometry analysis of PAH hydroxylates, formed in a simulator of the human gastrointestinal tract. J Chromatogr B.

[b31-ehp0113-000006] Van de Wiele TR, Verstraete W, Siciliano S (2004b). Polyaromatic hydrocarbon release from a soil matrix in the *in vitro* gastrointestinal tract. J Environ Qual.

[b32-ehp0113-000006] van Maanen JMS, Moonen EJC, Maas LM, Kleinjans JCS, van Schooten FJ (1994). Formation of aromatic DNA adducts in white blood cells in relation to urinary excretion of 1-hydroxypyrene during consumption of grilled meat. Carcinogenesis.

[b33-ehp0113-000006] van Schooten FJ, van Leeuwen FE, Hillebrand MJX, de Rijke ME, Hart AAM, van Veen HG (1990). Determination of benzo[*a*]pyrene diol epoxide-DNA adducts in white blood cell DNA from coke oven workers: the impact of smoking. J Natl Cancer Inst.

